# The use of dexmedetomidine and intravenous acetaminophen for the prevention of postoperative delirium in cardiac surgery patients over 60 years of age: a pilot study

**DOI:** 10.12688/f1000research.12552.2

**Published:** 2017-12-21

**Authors:** Ammu T. Susheela, Senthil Packiasabapathy, Doris-Vanessa Gasangwa, Melissa Patxot, Jason O’Neal, Edward Marcantonio, Balachundhar Subramaniam

**Affiliations:** 1 Department of Anesthesia, Critical Care and Pain Medicine, Beth Israel Deaconess Medical Center, Harvard Medical School, Boston, MA , 02215-5400, USA; 2 Department of Anesthesiology, Vanderbilt University Medical Center , 1215 21st Ave. S., Suite 5160 MCE NT , Nashville, Tennessee, 37232, USA; 3 Department of Medicine, Beth Israel Deaconess Medical Center, Harvard Medical School, Boston, MA , 02215-5400, USA

**Keywords:** delirium, dexmedetomidine, acetaminophen, cardiac surgery, coronary artery bypass grafting (CABG), propofol, cognitive assessments

## Abstract

**Background: **Delirium is associated with many negative health outcomes. Postoperative sedation and opioid administration may contribute to delirium. We hypothesize that the use of dexmedetomidine and Intravenous acetaminophen (IVA) may lead to reduced opioid consumption and decreased incidence of postoperative delirium. This pilot study aims to assess feasibility of using dexmedetomidine and IVA in cardiac surgical patients, and estimate the effect size for incidence and duration of delirium.

**Methods: **A total of 12 adult patients >60 years of age undergoing cardiac surgery were recruited and randomized into 4 groups: Propofol only (P), Propofol with IVA (P+A), Dexmedetomidine only (D), Dexmedetomidine with IVA (D+A). Preoperative baseline cognition and postoperative delirium was assessed daily until discharge. The feasibility was assessed by the number of patients who completed the study.

**Results: **All patients completed the study successfully. The total incidence of delirium in the study population was 42% (5/12):  67% (2/3) in the group P, and 67% (2/3) in the group D, 33% (1/3) in  D+A group and 0%(0/3)  P+A group. The incidence of delirium was 17% (1/6) in the group receiving IVA compared to 67% (4/6) that did not receive IVA. The mean range of duration of delirium was 0-1 days. One patient expired after surgery, unrelated to the study protocol. One patient in the D group experienced hypotension (systolic blood pressure <90 mm of Hg.)

**Conclusions: **The feasibility of performing a  project is ascertained by the study. Patients receiving IVA had lower incidence of delirium compared to patients not receiving IVA which suggests that IVA may have a role in reducing the incidence of delirium. A prospective randomized, placebo-controlled trial will be the next step in investigating the role of dexmedetomidine and IVA in reducing the incidence of delirium.

## Introduction

Delirium is defined as a change in mental status, characterized by acute onset and fluctuating course, inattention, disorganized thinking, and altered level of consciousness ^1, 2^. Delirium increases the risk of mortality, readmissions, and accelerated cognitive decline ^2– 6^. Around 158 billion dollars of national healthcare cost is attributable to delirium ^7^. The incidence of delirium in cardiac surgery is 11–46% ^2^. Delirium is preventable in 30–40% of the cases ^2^. Some of the modifiable risk factors for delirium include the choice of analgesic and sedatives ^8^.

Currently used sedatives, like midazolam, act via GABA receptors and release deliriogenic mediators ^9^. Dexmedetomidine is an alpha 2 adrenergic agonist, with no interaction with GABA receptors.

A recent study by Li *et al.* in 285 elderly cardiac surgical patients evaluated dexmedetomidine versus propofol and showed no difference in the incidence of delirium ^10^. A major limitation of this study is that CAM- ICU is limited in delirium assessments and may miss the diagnosis. There are other tools that could help assess cognitive assessment more efficiently. A meta-analysis suggested that the use of dexmedetomidine for sedation in cardiac surgery patients may reduce the incidence of delirium ^9^. The first study in the meta-analysis was a retrospective study by Corbett SM *et al*. that does not mention how delirium was assessed in individual hospitals ^11^. The second study by Shehabi Y *et al.* was not powered adequately and used CAM-ICU for assessing delirium both in intubated and extubated patients which have reduced sensitivity for delirium in verbal patients and could have underestimated the incidence of delirium ^12^. The third study by Maldonado JR et al. of recruited118 patients and 90 patients were finally analyzed. 28 randomized patients were excluded for protocol violations which introduce a significant selection bias ^13^. It also showed a 94% reduction in the incidence of delirium and this effect size is almost implausibly large and has not been seen in other studies. The final study by Dasta JF *et al.* was not designed to identify patients with delirium with any specific tools such as CAM and thus could have gross underestimated delirium incidence ^14^. The use of IV acetaminophen (IVA) has been shown in multiple studies to reduce the amount of opioids consumed by patients undergoing surgeries ^4, 15^. IVA has never been studied in the context of cardiac surgery and delirium prevention. So, we hypothesized that the use of dexmedetomidine and acetaminophen would provide adequate analgesia, leading to a decreased opioid consumption and thus decreased incidence of delirium.

## Methods

### Purpose

This is a pilot trial which ran from November 2013 to November 2014 to assess the feasibility of using IV dexmedetomidine and acetaminophen in the cardiac surgical intensive care unit. The study also aimed to obtain effect size estimates for primary study outcomes which will help power an ongoing large scale randomized trial (NCT02546765, registered on 13 ^th^ January 2015).

### Study design

This is a single-centered, double-blinded, prospective, randomized controlled pilot trial.

### Study population

**Figure 1.**
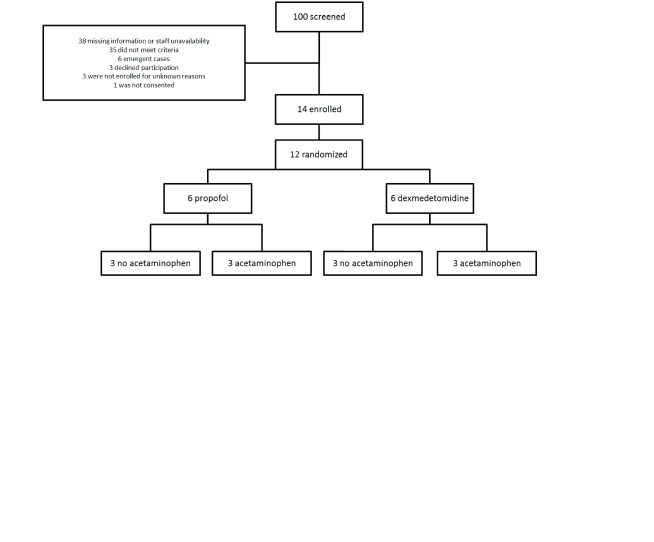
Consort diagram.

A homogenous set of patients were chosen for this feasibility study. Patients who are 60 years of age or older who were undergoing coronary artery bypass grafting (CABG), and/or valve surgery were included in the study. Exclusion criteria were preoperative left ventricular ejection fraction < 30%, emergent and percutaneous procedures, aortic surgeries, preexisting cognitive impairment, recent seizures, patients on medications for cognitive decline, serum creatinine > 2 mg%, liver dysfunction, known history of alcohol or drug abuse, and hypersensitivity to any of the study drugs. Patients who might not get extubated in a reasonable amount of time (hence the EF, aortic surgery exclusion, etc) were excluded. Since Q6H IV acetaminophen was given, any clinical situation that can potentially set up patients for drug toxicity etc. were avoided. ( Figure 1)

### Randomization

Fourteen patients were recruited (the initial 2 patients to help train the investigators in the use of cognitive assessments). The remaining twelve patients were randomized into the following 4 groups, containing 3 patients each (1:1:1:1 allocation). ( Table 1)

**Table 1.** Table 1 provides all different groups in the study.

Group *	Sedation	Acetaminophen
**Group 1 (P)**	Propofol	No
**Group 2 (P+A)**	Propofol	Yes
**Group 3 (D)**	Dexmedetomidine	No
**Group 4 (D+A)**	Dexmedetomidine	Yes

*All groups will receive bolus doses of IV opioids (morphine and hydromorphone) as needed for breakthrough pain. I.V. Acetaminophen will be given to group 2 and group 4 every 6 hours for 48 hours, postoperatively.

Each patient was allotted a randomization number based on which the pharmacist assigned the study medications.

### Study drug administration

Peri-operative anesthetic management was administered according to standard of care. Intra-operative propofol infusion was administered at the clinician’s discretion for patients in propofol group. In the post-operative period, propofol infusion was titrated to 25–100 µg/kg/min. In the dexmedetomidine group, dexmedetomidine infusion was given after chest closure at a dose of 0.1–1.0 µg/kg/hr. The medications for sedation were continued in the post-operative period until extubation or for a minimal duration of 6 hours. For patients randomized to IVA group, IVA 1 gram was given every 6 hours for the first 48 hours postoperatively up to a total of eight doses.

### Patient assessment

Cognitive assessments were conducted using standard battery used for Mini Mental (MMSE) and Confusion Assessment Method (CAM).

### Outcomes of the study

The primary outcome measured was the proportion of patients who completed the protocol successfully, which reflects the feasibility of the study. The incidence of delirium was measured to obtain effect size estimates for future studies. Secondary outcomes included hypotension, duration of delirium, breakthrough analgesic requirements, ICU days, and length of hospital stay.

## Results

The baseline characteristics of the patients were comparable in all groups. The total incidence of delirium was 42% (5/12). The incidence of delirium was 67% (2/3) with propofol and 67% (2/3) with dexmedetomidine. Dexmedetomidine+Acetaminophen group had an incidence of 33% (1/3). The Propofol+Acetaminophen group had no occurrence of delirium. Interestingly, only 17% (1/6) of the subjects who received IVA were diagnosed with delirium compared to 67% (4/6) in the group who did not receive IVA. ( Table 2) Also, the incidence of delirium was 33% (2/6) in the propofol groups as compared to 50% (3/6) in the dexmedetomidine groups. The mean duration of delirium ranged from 0 to 1 day. ( Table 3) Secondary outcomes were similar between the groups. One patient expired after surgery, unrelated to the study protocol. One patient in the dexmedetomidine group experienced a significant hypotension with systolic blood pressure <90 mm of Hg.

**Table 2.** Table 2 provides the incidence of delirium and secondary outcomes in the groups receiving propofol, dexmedetomidine, acetaminophen, and no acetaminophen respectively.

	Propofol	Dexmedetomidine	Acetaminophen	No Acetaminophen
**Incidence of Delirium** **% (n/N)**	33.3 (2/6)	50 (3/6)	16.7 (1/6)	66.7 (4/6)
**Mean Delirium duration** **[n days]**	0.5	1	0.5	1
**Opioid consumption** mean [SD] *	8.2 [2.02]	11.46 [4.2]	7.54 [2.15]	12.12 [4.06]
**Time to extubation (mins)** **mean [SD]**	410.67 [320.25]	439 [239.32]	499.5 [304.63]	350.17 [232.66]
**Length of stay (days)** **mean [SD]**	5.67 [0.82]	8.5 [6.5]	7.83 [6.52]	6.33 [1.97]
**Adverse events**	0	1	0	1
**Dexmedetomidine dose** mean [SD] **	N/A	0.95 [0.36]	N/A	N/A
**Propofol dose** mean [SD] ***	43.33 [5.16]	N/A	N/A	N/A

*Opioid consumption is expressed in hydromorphone equivalent

**Mean dexmedetomidine dose is expressed as infusion rate of mcg/kg/hr

***Mean propofol dose is expressed as infusion rate of mcg/kg/min

**Table 3.** Table 3 provides the incidence of delirium and secondary outcomes in the 4 groups in the study namely propofol, propofol + acemaninophen, dexmedetomidine, dexmedetomidine + acetaminophen.

	Propofol	Propofol + Acetaminophen	Dexmedetomidine	Dexmedetomidine + Acetaminophen
**Incidence of Delirium** **% (n/N)**	66.6 (2/3)	0 (2/3)	66.6 (2/3)	33.3 (1/3)
**Mean Delirium duration** **[n days]**	1	0	1	1
**Opioid consumption** mean [SD] *	2.84 [1.95]	2.63 [2.2]	5.24 [5.3]	2.4 [2.2]
**Time to extubation (mins)** **mean [SD]**	213.67 [42.83]	607.67 [371.66]	486.67 [278.55]	391.33 [242.43]
**Length of stay (days)** **mean [SD]**	6 [0]	5.33 [1.15]	6.67 [3.06]	10.33 [9.29]
**Adverse events**	0	0	0	1

*Opioid consumption is expressed in hydromorphone equivalent

The datasets used and/or analyzed during the current study. No statistical evaluation was done due to the small sample size Click here for additional data file. Copyright: © 2017 Susheela AT et al. 2017 Data associated with the article are available under the terms of the Creative Commons Zero "No rights reserved" data waiver (CC0 1.0 Public domain dedication).

## Discussion

The use of IV dexmedetomidine and acetaminophen in the cardiac surgical patients was feasible. The study protocol was easily incorporated into patient care. All the enrolled patients completed the study protocol. There were no instances where the implementation of the study protocol was abandoned by the physicians. Sedation provided was useful. Apart from the incidence of hypotension in one patient with dexmedetomidine use, no other adverse events recorded directly related to the intervention.

No other statistical evaluation was done due to the small sample size. The overall incidence of delirium in our trial, at 42%, is close to previous reports ^2^. The incidence of delirium in patients receiving IVA was 17% compared to 67% in the other group suggesting that IVA may be beneficial in reducing the incidence of delirium following cardiac surgery. The pilot study led to a larger ongoing trial (NCT02546765).

A major limitation of our study is the small sample size, but this was a feasibility trial to study effect size for the design of future larger studies. Also, there was no blinding for the choice of sedatives used in the patients. The assessors were blinded to acetaminophen administration.

A multi-center randomized, controlled trial will be the next step in investigating the role of dexmedetomidine and IVA in reducing the incidence of delirium.

## Data availability

The data referenced by this article are under copyright with the following copyright statement: Copyright: © 2017 Susheela AT et al.

Data associated with the article are available under the terms of the Creative Commons Zero "No rights reserved" data waiver (CC0 1.0 Public domain dedication). 

F1000Research: Dataset 1. The datasets used and/or analyzed during the current study. No statistical evaluation was done due to the small sample size., 10.5256/f1000research.12552.d180828 ^16^

## Ethics and consent

This study has been approved by the Committee on Clinical Investigations at Beth Israel Deaconess Medical Center (IRB Protocol #: 2013-P-000149). Informed consent was obtained for all subjects prior to initiation of study procedures. The study was conducted from 13 ^th^ November 2013 to 9 ^th^ April 2015.
