# Supplementary material for: The use of dexmedetomidine and intravenous acetaminophen for the prevention of postoperative delirium in cardiac surgery patients over 60 years of age: a pilot study
Source: F1000Res. 2017 Dec 21;6:1842. Originally published 2017 Oct 16. [Version 2] doi: 10.12688/f1000research.12552.2 (PMC5754745; doi:10.12688/f1000research.12552.2)
Supplement: Peer review history

# Peer review: Referee response for version 2

**DOI:** 10.5256/f1000research.14687.r29300

**Published:** 2018-01-15

**Authors:** George Djaiani

**Affiliations:** 1 Department of Anesthesia and Pain Management, Toronto General Hospital, University Health Network, University of Toronto, Toronto, ON, Canada

The authors report results of a pilot study looking at postoperative administration of acetaminophen in reducing the incidence of postoperative delirium (POD) after cardiac surgery in elderly patients.

 I believe that the authors are asking an important question whether adequate or superior pain relief is associated with reduced POD rates after cardiac surgery.

  Concerns:

1.  It is not clear to me why this pilot study is referred to as a feasibility trial. Both dexmedetomidine and acetaminophen are used routinely in many cardiac centres. Please comment. 
2. I would strongly advise against quoting outcome measures as percentages of patients, you have 3 patients per group, e.g., quoting 2/3 (67%) is misleading.
3. The authors found that DEX group received twice as much opioids compared to the other groups. It is in contrary with previous studies identifying opioid sparing effects of DEX. (e.g. Anaesthesiology 2016; 124(2): 362-8; Lancet 2016; 15;388(10054): 1893-1902), however, it could be contributed to small sample size and the DEX administration protocol. Please comment.
4. If I understood correctly DEX was given until extubation or for 6 hours postoperatively. Not a standard administration practice for DEX. Please comment on rational for this design.
5. With respect to pain relief after cardiac surgery, spinal/epidural analgesia has been associated with superior pain relief and significant opioid sparing effects, however, there are no studies that have shown a reduction in delirium rates with either of these techniques. Please comment if acetaminophen may have some intrinsic properties other than analgesia in reducing POD after cardiac surgery.  

I have read this submission. I believe that I have an appropriate level of expertise to confirm that it is of an acceptable scientific standard.

# Peer review: Referee response for version 2

**DOI:** 10.5256/f1000research.14687.r29246

**Published:** 2018-01-04

**Authors:** Michael S. Avidan

**Affiliations:** 1 Department of Anesthesiology, Washington University School of Medicine, Saint Louis, MO, USA

The investigators have addressed my concerns.

I have read this submission. I believe that I have an appropriate level of expertise to confirm that it is of an acceptable scientific standard.

# Response

**Published:** 2018-01-05

**Authors:** Ammu Susheela

**Affiliations:** Department of Anesthesiology, Beth Israel Deaconess Medical Center, USA

Thank you

# Peer review: Referee response for version 2

**DOI:** 10.5256/f1000research.14687.r29299

**Published:** 2017-12-28

**Authors:** Yahya Shyehabi

**Affiliations:** 1 Faculty of Medicine, Nursing and Health Sciences, Monash University, Clayton, VIC, Australia

Although this study is considered a pilot trial, the small number of patients 12 divided into 4 groups is a serious flaw in methodology. The interpretation of the data is completely meaningless not even as a feasibility study.

 I believe this study should not be more than a run-in phase for a proper pilot with reasonable numbers.

 I do not recommend publication in F1000.

 Thanks.

I have read this submission. I believe that I have an appropriate level of expertise to state that I do not consider it to be of an acceptable scientific standard, for reasons outlined above.

# Peer review: Referee response for version 2

**DOI:** 10.5256/f1000research.14687.r29245

**Published:** 2017-12-22

**Authors:** Nicolai Goettel

**Affiliations:** 1 Department of Anesthesia, University of Basel, Basel, Switzerland

I do not have further comments to make to this revision of the article regarding the pilot study. I wish the investigators good luck with the follow-up (single- or multicenter) trial.

I have read this submission. I believe that I have an appropriate level of expertise to confirm that it is of an acceptable scientific standard.

# Response

**Published:** 2017-12-22

**Authors:** Ammu Susheela

**Affiliations:** Department of Anesthesiology, Beth Israel Deaconess Medical Center, USA

Thank you. We appreciate your comments.

# Peer review: Referee response for version 1

**DOI:** 10.5256/f1000research.13591.r27502

**Published:** 2017-11-27

**Authors:** Michael S. Avidan

**Affiliations:** 1 Department of Anesthesiology, Washington University School of Medicine, Saint Louis, MO, USA

This is an interesting and important feasibility study, which seeks to determine whether it is possible to conduct a (large) study evaluating various combinations of sedation/analgesia post cardiac surgery, with the intention of preventing postoperative delirium. In general, with a very small feasibility study, the investigators demonstrate that conducting such a study might be possible. However this study has important constraints.

1. The investigators state, “The mean duration of delirium was 0-1 days.” This appears to be a range.
2. The investigators conclude, “The feasibility of performing a large-scale project is ascertained by the study.” I would rather argue that the feasibility of performing a project is ascertained. Whether or not a large-scale project is feasible is not necessarily established.
3. Contrary to the investigators, I do not believe that a 12 patient study with four groups supports the inference “that IVA may have a role in reducing the incidence of delirium.” This is over-interpretive of very limited data.
4. I do not agree that using the CAM is a major limitation in delirium research (as the investigators suggest that it might lack sensitivity). I do agree that this might apply to the CAM-ICU.
5. More efficient tools might not necessarily be more sensitive.
6. I am not convinced that a 12 patient pilot trial will produce sufficiently precise estimates to power a large RCT appropriately. Although the absolute difference between proportions (delirium incidence) was 50% (67% minus 17%), the 95% confidence interval for this difference is -13% to 82%. Thus, it is not clear based on this small study what value should be used (estimated difference in delirium incidence between the groups [acetaminiphen and placebo]) for the sample size calculation.
7. From the registration site (for NCT02546765), it appears that 120 patients will be included in the (large) trial. This number is likely to be much too small to answer the research question with sufficient precision.
8. The reasons for the exclusion criteria are not clear.
9. With the modified factorial design, was interaction between interventions considered? Specifically, both dexmedetomidine and acetaminophen might have an impact on the outcome of interest (delirium).
10. The (secondary) results of the study were as follows: “The Propofol+Acetaminophen group had no occurrence of delirium. Interestingly, only 17% (1/6) of the subjects who received IVA were diagnosed with delirium compared to 67% (4/6) in the group who did not receive IVA.” The investigators should be cautious about over-interpreting these findings, or using these estimates to justify sample size for a large trial. The problem with this would be that 67% is a very high incidence, and is not likely to reflect the incidence with current standard of care (i.e., propofol or dexmedetomidine sedation, without intravenous acetaminophen). Similarly, the incidence of 17% is actually likely to be pretty close to the current delirium incidence in the type of patients who were enrolled to this study (but who receive standard of care).

I have read this submission. I believe that I have an appropriate level of expertise to confirm that it is of an acceptable scientific standard, however I have significant reservations, as outlined above.

# Response

**Published:** 2017-12-13

**Authors:** Ammu Susheela

**Affiliations:** Department of Anesthesiology, Beth Israel Deaconess Medical Center, USA

Dear Dr. Micheal Avidan,

Thank you for reviewing the paper. We have thoroughly reviewed the comments. We are addressing below, point-by-point all the suggestions. 

1. The investigators state, “The mean duration of delirium was 0-1 days.” This appears to be a range. **Thank you. Yes. We reported the mean duration as a range.**
2. The investigators conclude, “The feasibility of performing a large-scale project is ascertained by the study.” I would rather argue that the feasibility of performing a project is ascertained. Whether or not a large-scale project is feasible is not necessarily established. **Thank you. We agree that only the feasibility of performing a project is ascertained.**
3. Contrary to the investigators, I do not believe that a 12 patient study with four groups supports the inference “that IVA may have a role in reducing the incidence of delirium.” This is over-interpretive of very limited data. **Thank you. We agree this is a pre-pilot study indeed.  It was a feasibility project and we certainly do not want to overinterpret.  Thus, we wrote “IVA may be beneficial in reducing the incidence of delirium following cardiac surgery. ”  Since this is a feasibility study we agree that a larger study is needed to draw a concrete conclusion.**
4. I do not agree that using the CAM is a major limitation in delirium research (as the investigators suggest that it might lack sensitivity). I do agree that this might apply to the CAM-ICU. **Thank you. Agreed.  **
5. More efficient tools might not necessarily be more sensitive. **Thank you. Agreed.**
6. I am not convinced that a 12 patient pilot trial will produce sufficiently precise estimates to power a large RCT appropriately. Although the absolute difference between proportions (delirium incidence) was 50% (67% minus 17%), the 95% confidence interval for this difference is -13% to 82%. Thus, it is not clear based on this small study what value should be used (estimated difference in delirium incidence between the groups [acetaminiphen and placebo]) for the sample size calculation. **Thank you. We planned this pilot study as a means to assess the feasibility of performing a large-scale project.  However, there is no other preexisting data to power the next larger trial.  Since this pilot study showed a 66% reduction in the incidence of delirium, for the power calculation, a 50% reduction in the delirium incidence was used.  Each group needed 56 patients (alpha=0.05 and 1-Beta=0.80).**
7. From the registration site (for NCT02546765), it appears that 120 patients will be included in the (large) trial. This number is likely to be much too small to answer the research question with sufficient precision. **Thank you.  A multicenter trial is definitely required to answer with precision.  However, it will be a major leap to go from a pilot study to a multicenter trial.  Thus, we chose to do the second step of testing in a larger sample size with what is known so far.  Depending on what this study shows, the obvious next step will be a larger multicenter trial.**
8. The reasons for the exclusion criteria are not clear. **Thank you. We chose a homogenous set of patients to determine the drug efficacy.  We wanted to avoid patients who might not get extubated in a reasonable amount of time (hence the EF, aortic surgery exclusion, etc).  As we chose to give Q6H IV acetaminophen, we wanted to avoid any clinical situation that can potentially set up patients for drug toxicity etc.  Preexisting cognitive dysfunction, etc. could muddle the picture in a 120 patient study.  In a larger multicenter trial if and when launched, the heterogeneity can be accounted for and thus need not necessarily be an exclusion criteria.**
9. With the modified factorial design, was interaction between interventions considered? Specifically, both dexmedetomidine and acetaminophen might have an impact on the outcome of interest (delirium). **Thank you. Yes, these interactions will be analyzed in the ongoing trial**
10. The (secondary) results of the study were as follows: “The Propofol+Acetaminophen group had no occurrence of delirium. Interestingly, only 17% (1/6) of the subjects who received IVA were diagnosed with delirium compared to 67% (4/6) in the group who did not receive IVA.” The investigators should be cautious about over-interpreting these findings, or using these estimates to justify sample size for a large trial. The problem with this would be that 67% is a very high incidence, and is not likely to reflect the incidence with current standard of care (i.e., propofol or dexmedetomidine sedation, without intravenous acetaminophen). Similarly, the incidence of 17% is actually likely to be pretty close to the current delirium incidence in the type of patients who were enrolled to this study (but who receive standard of care). **Thank you for the caution.  We agree with all the comments.  **

# Peer review: Referee response for version 1

**DOI:** 10.5256/f1000research.13591.r27423

**Published:** 2017-11-09

**Authors:** Nicolai Goettel

**Affiliations:** 1 Department of Anesthesia, University of Basel, Basel, Switzerland

In this single center randomized controlled trial (RCT), Susheela and coworkers investigate the use of dexmedetomidine and intravenous acetaminophen for the prevention of postoperative delirium in elderly patients undergoing cardiac surgery. This pilot study primarily aimed to assess the feasibility of a larger RCT with identical interventions.

 As a pilot project, the study is methodically sound. Overall, the manuscript is well-written and pleasant to read. The authors applied CONSORT criteria which is a definite plus.

 Postoperative delirium has specific features that may be related to surgery (and anesthesia) and has a probable neuroinflammatory component. I therefore do not agree with your statement that 30-40% of delirium cases could be prevented, at least not in your sample of patients undergoing cardiac surgery. So far, multicomponent interventions to prevent delirium have only been proven efficacious in a general population of elderly hospitalized patients. ^1^

 Please also discuss the opioid-sparing effects of intraoperative dexmedetomidine. Next to the missing interaction with GABA receptors, this might be an additional mechanism for delirium prevention when using dexmedetomidine.

 Please discuss the reasons to exclude patients with preexisting cognitive impairment from your study. Preoperative cognitive impairment was found to be one of the most important risk factors for postoperative delirium (odds ratio ranged from 6.3 (95% CI 2.9 to 13.7) up to 11.5 (95% CI 6.1 to 20.1)). ^2 ^By excluding these patients (also in the follow-up RCT), you likely decrease the number of patients affected by the primary outcome measure (postoperative delirium) in your study. It would be interesting to investigate the effects of dexmedetomidine and intravenous acetaminophen in these high-risk patients.

 I find it difficult to blind the intravenous administration of propofol and dexmedetomidine. At best, you may try to conceal the study drug from patient, surgeon, ICU caregivers, and investigators by using opaque syringes and lines. Was study drug administration really double-blind?

 Was there any kind of standardization in anesthesiologic management? If yes, state drugs and dosage.

 At what frequency did you assess patients for postoperative delirium in the ICU/on the ward? Who assessed the patients?

 I wish the investigators of this pilot project the best of luck with the larger ongoing trial!

 References

1. Inouye SK, Bogardus ST Jr, Charpentier PA, et al. A multicomponent intervention to prevent delirium in hospitalized older patients. N Engl J Med 1999; 340: 669–76.
2. National Institute for Health and Care Excellence. NCGC National Clinical Guideline Centre. Delirium: diagnosis, prevention and management. http://www.nice.org.uk/guidance/cg103/evidence/full-guideline-134653069.

I have read this submission. I believe that I have an appropriate level of expertise to confirm that it is of an acceptable scientific standard.

# Response

**Published:** 2017-11-10

**Authors:** Ammu Susheela

**Affiliations:** Department of Anesthesiology, Beth Israel Deaconess Medical Center, USA

Dear Nicolai Goettel,

We sincerely thank you for the opportunity to address the comments of our manuscript. We have thoroughly reviewed the comments. In this letter, we address, point-by-point, all the recommendations. 

We thank you for the review.

Response

In this single-center randomized controlled trial (RCT), Susheela and coworkers investigate the use of dexmedetomidine and intravenous acetaminophen for the prevention of postoperative delirium in elderly patients undergoing cardiac surgery. This pilot study primarily aimed to assess the feasibility of a larger RCT with identical interventions.

As a pilot project, the study is methodically sound. Overall, the manuscript is well-written and pleasant to read. The authors applied CONSORT criteria which is a definite plus.

**Thank you**

Postoperative delirium has specific features that may be related to surgery (and anesthesia) and has a probable neuroinflammatory component. I, therefore, do not agree with your statement that 30-40% of delirium cases could be prevented, at least not in your sample of patients undergoing cardiac surgery. So far, multicomponent interventions to prevent delirium have only been proven efficacious in a general population of elderly hospitalized patients.1

**Thank you. These estimates are from the pilot study result.  The ongoing study will inform us better about the effect size.**

Please also discuss the opioid-sparing effects of intraoperative dexmedetomidine. Next, to the missing interaction with GABA receptors, this might be an additional mechanism for delirium prevention when using dexmedetomidine.

**Thank you. Agreed. It is possible that dexmedetomidine can reduce the analgesic requirements. The ongoing study will inform us of this effect size as well due to the crossover design.**

Please discuss the reasons to exclude patients with preexisting cognitive impairment from your study. Preoperative cognitive impairment was found to be one of the most important risk factors for postoperative delirium (odds ratio ranged from 6.3 (95% CI 2.9 to 13.7) up to 11.5 (95% CI 6.1 to 20.1)).2 By excluding these patients (also in the follow-up RCT), you likely decrease the number of patients affected by the primary outcome measure (postoperative delirium) in your study. It would be interesting to investigate the effects of dexmedetomidine and intravenous acetaminophen in these high-risk patients.

**Thank you. As this is the first study of this size, we would like to get a homogenous group examined first and definitely the next step is to do the patients with preexisting cognitive dysfunction**.

I find it difficult to blind the intravenous administration of propofol and dexmedetomidine. At best, you may try to conceal the study drug from patient, surgeon, ICU caregivers, and investigators by using opaque syringes and lines. Was study drug administration really double-blind?

**Thank you. We only double-blinded the Acetaminophen arms.**

Was there any kind of standardization in anesthesiologic management? If yes, state drugs and dosage.

**Cardiac surgery is the most protocolized intraoperative setting. Patients were induced with 500mcg of IV fentanyl, 50-100mg of Propofol, and endotracheal intubation was facilitated with 1mg/kg intravenous rocuronium.  Anesthesia was maintained with isoflurane (0.6-1.0 MAC) in 100% oxygen with intermittent fentanyl** **up to** **a maximum of additional 500 mcg of fentanyl and intermittent rocuronium.  All patients were taken intubated to the ICU**

****

At what frequency did you assess patients for postoperative delirium in the ICU/on the ward? Who assessed the patients?

**Assistants trained from Edward Marcantonio’s lab (with initial half a day training session, witnessing the assessments for a few patients and also calibration with gold standard assessor).** **Postoperative** **delirium was assessed by a trained study team member with the help of cognitive assessments such as MMSE, CAM-ICU, and CAM starting from the day after the surgery. If cognitive assessments are negative for 3 consecutive days (i.e days 5, 6 & 7), assessments were completed every other day until the** **date** **of discharge.**

I wish the investigators of this pilot project the best of luck with the larger ongoing trial!

**Thank you**
